# Of Chains and Rings: Synthetic Strategies and Theoretical Investigations for Tuning the Structure of Silver Coordination Compounds and Their Applications

**DOI:** 10.3390/ma3053407

**Published:** 2010-05-25

**Authors:** Tünde Vig Slenters, Jorge L. Sagué, Priscilla S. Brunetto, Stefanie Zuber, Antoine Fleury, Laurent Mirolo, Adeline Y. Robin, Markus Meuwly, Oliver Gordon, Regine Landmann, Alma U. Daniels, Katharina M. Fromm

**Affiliations:** 1 Department of Chemistry, University of Fribourg, Chemin du Musée 9, CH-1700 Fribourg, Switzerland; 2 Department of Chemistry, University of Basel, CH-4056 Basel, Switzerland; 3 Infection Biology, University Hospital Basel, CH-4056 Basel, Switzerland; 4 Lab of Biomechanics & Biocalorimetry, Faculty of Medicine, University of Basel, CH-4056 Basel, Switzerland

**Keywords:** coordination polymer networks, silver coordination compounds, molecular rings, helical chains, structure engineering, metal-organic frameworks, antimicrobial coatings, implant materials

## Abstract

Varying the polyethyleneglycol spacer between two (iso)-nicotinic groups of the ligand systems, a large structural variety of silver coordination compounds was obtained, starting with zero-dimensional ring systems, via one-dimensional chains, helices and double-helices to two-dimensional polycatenanes. Theoretical calculations help to understand their formation and allow predictions in some cases. These structures can be tuned by careful design of the ligand, the use of solvent and the counter ions, influencing also other important properties such as light stability and solubility. The latter is important in the context of biomedical applications, using silver compounds as antimicrobial agents.

## 1. Introduction

Coordination compounds forming infinite motifs in one, two or three dimensions have been investigated over the past years for several reasons: structure prediction, tuning of chemical and physical properties, as well as applications as materials [1,2,3,4,5,6,7,8]. One of the main reasons is certainly the challenge of crystal engineering, being able to predict a solid state structure for a given combination of building blocks, based on metal ions, ligands and, eventually, counter ions and co-crystallizing solvent [9]. This can lead to ideal combinations of properties of the different building blocks, which are useful in applications such as in host-guest chemistry, catalysis, magnetic and optical applications, to name a few. Yet, despite the enormous efforts in terms of ligand design, choice of metal ions and reaction conditions, chemists are still confronted with surprises in the outcome of the final products and their structure [10].

Having studied a number of silver coordination compounds and polymers for their antimicrobial properties as well as their capacity to form silver nanoparticles [11,12], we wish to highlight here a panoply of silver coordination compounds based on a series of ligands in which only the spacer between the main coordinating units is changed. The differences in structure type are presented here as a review together with some missing puzzle pieces in order to complete the picture of the variety of structures available in this way.

## 2. Results and Discussion

**Scheme 1:**
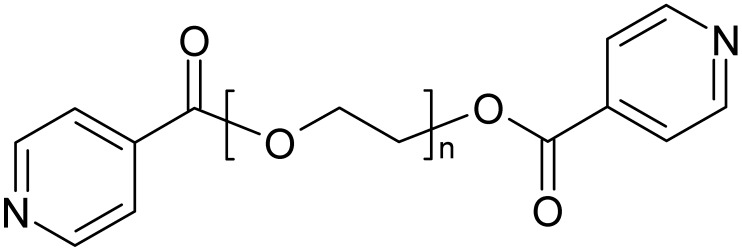
Ligands used in this work, based on isonicotinic acid end groups on short polyethyleneglycoles (L1, L2) (n = 1 ➔ L1, n = 2 ➔ L2).

The two ligands, derived from mono-, respectively bis-ethyleneglycol and two isonicotinic acid end groups, L1 and L2, were used for the following studies (Scheme 1). These ligands possess a flexible backbone with O-donor functions, and N-donor atoms at both ends, allowing in principle for different metal ions to be coordinated at the individual Lewis basic sites. Furthermore, the ligands are built up from biocompatible building blocks and quite easily accessible in synthesis. With the idea of antimicrobial properties in mind, we started to investigate the coordination of the ligands toward silver ions and were quite surprised by the panoply of different structures that are obtained based on these ligands.

### 2.1. Compounds with L1

**Figure 1:**
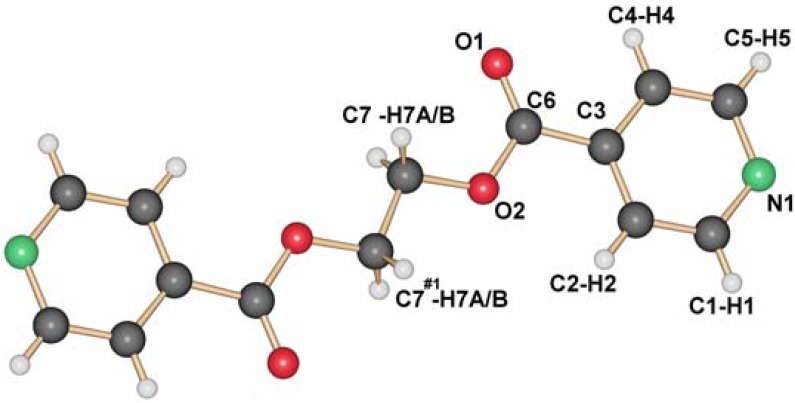
Molecular structure of L1.

As shown previously by us [13], the ligand L1 crystallizes adapting the anti conformation around the ethyl group as depicted in Figure 1. This seems to be its preferential conformation in the solid state, although calculations for the ligand in the gas phase show that the rotation around the C–C bonds should occur easily at room temperature. Yet, most of the silver coordination compounds obtained with this ligand have it in this orientation.

**Figure 2:**
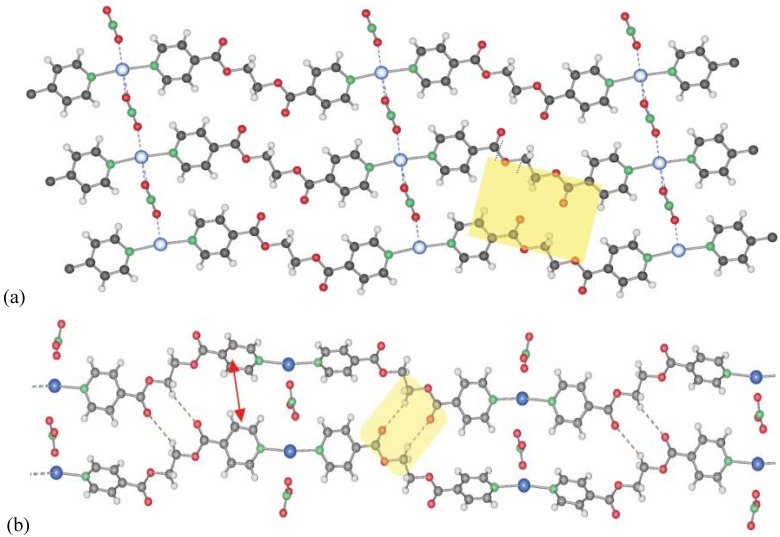
Two polymorphs of [Ag(L1)NO_3_]: **1** (a) and **2** (b), varying in the orientation of the ligand molecules to each other (blue: Ag; red: O; green: N; grey: C; white: H; yellow: H-bonding motifs, H-bonds for **1** are in the range of *d*(D–A) 3.457(4)-3.543(4) Å; **2**: 3.5807(7)Å).

For silver nitrate and ligand L1 as starting material [14], a large number of coordination polymers based on one-dimensional chain structures were obtained. In these compounds, the silver ions are generally coordinated by two ligand molecules each, while the nitrate ions play the role as bridging units within or between the chains. Apart from a two-dimensional compound based on the composition {[Ag_2_(NO_3_)_2_(L1)]}, the composition [Ag(L1)NO_3_] allows two polymorphs **1** and **2** (Figure 2), which differ more or less only in the orientation of the ligand molecules with respect to each other [15].

Compounds **1** and **2** are obtained from different solvents, namely acetonitrile for compound **1** (space group *P*2_1_/n, *a* = 11.6742(8), *b* = 11.0728(6), *c* = 12.1813(9)Å, *β* = 99.016(6)°) and THF/water mixture for compound **2** (space group *P*-1, *a* = 6.159(1), *b* = 8.895(3), *c* = 14.439(3)Å, *α* = 93.15(2), *β* = 99.90(2), *γ* = 91.43(2)°). Single crystals of these coordination polymers are then formed by slow diffusion processes in H-formed tubes, where in each arm of the H-tube, one of the starting materials-ligand on one hand and silver nitrate on the other is placed. Slow diffusion through solvent or solvent mixtures then allows single crystalline material to be obtained.

**Figure 3:**
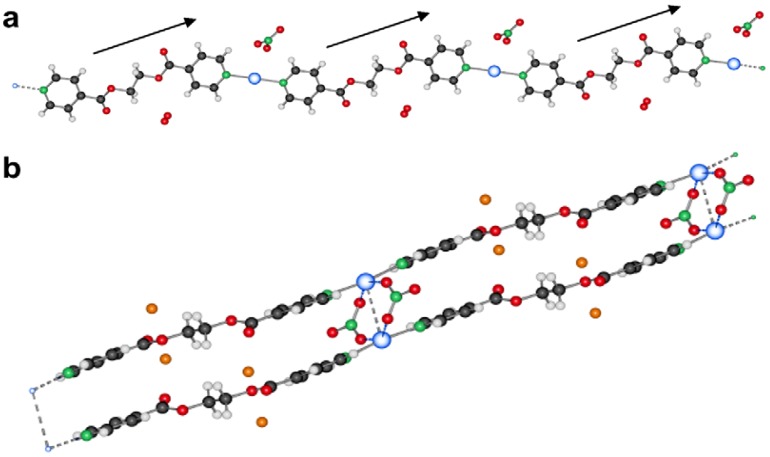
(a) Simple chain motif of compound **3**; (b) detailed double chain motif of **3** (same color codes as Figure 2, orange in b: water molecules).

At first sight, compound **3** is identical to compound **1**, however compound **3** (Figure 3a) is obtained similar to **2** from THF/water, but on the water side of the H-tube, and contains two water molecules per silver ion to give compound **3** as [Ag(L1)NO_3_](H_2_O)_2_ [16]. The main difference to compound **1** is the formation of pairs of chains with very close Ag–Ag contacts and two additional water molecules.

The water-free compound **4** [Ag(L1)NO_3_] [16] is obtained as polycrystalline solid by direct mixing of ligand L1 and silver nitrate. As in compound **3**, the pairs of chains are observed. Compounds **3** and **4** are structurally related to each other via a clay-like behavior in terms of reversible loss and uptake of water molecules. The uptake of two water molecules per unit cell is reflected in a unit cell increase from **3** to **4** of about 40 Å^3^ and a shift of the chains parallel to each other. During this process, the single crystals of **3** deteriorate into the polycrystalline powder.

**Figure 4:**
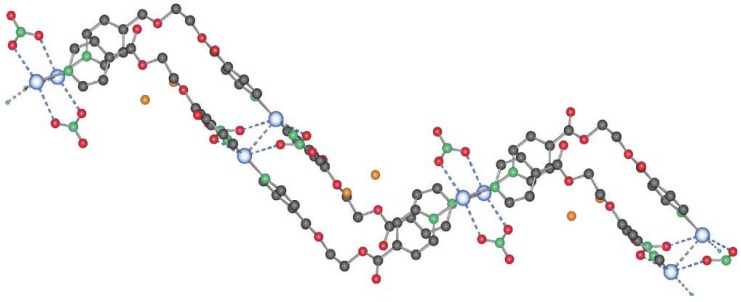
Double-chain structure of compound **5** (same color code as previously).

The only compound identified by us having the ligand L1 in a gauche conformation in the final coordination polymer is [Ag(L1)NO_3_](H_2_O), **5** [14]. It is also formed by pairs of chains as in **3** and **4**, but now the conformation of L1 is extremely different. Among all compounds, **5** was the most rare to obtain (Figure 4).

The nitrate anion in compounds **1**–**5** is very flexible in its bridging mode and strength. It is therefore able to act as bridging ligand between two silver atoms as in **1** or **2** in which it links several chains together. It also shows its ability to support a metal–metal contact in **3**, **4** and **5**. Its coordination strength can be tuned by the number of hydrogen bonds in which it can be involved with, for instance water molecules. Thus, the Ag–O(nitrate) distances increase with the number of co-crystallizing solvent. Solvation of the cations and anions of AgNO_3_ also plays a role in final Ag–NO_3_ distances in the products’ crystal structure. The influence of the solvent has been discussed in detail by us previously for the different compounds **1**–**5**. For example, we have shown that the Ag–O(NO_3_)-distances in our compounds **1**–**5** differed and depended on the solvents used during crystallization. The better the initial solvent for AgNO_3_, the longer the final Ag–O in the solid state. The solvent contribution can thus be resumed as the different solvation of the reagents by the solvent in the crystallization process. In some cases, the interactions between solvent molecules and reagents are reflected in the solid state, like in **1** and **2**, and in other cases, solvent molecules co-crystallize, as in **3**, and **5**.

In order to investigate the influence of other counter ions in search for possible other examples with the ligand L1 in gauche conformation, we now present two new examples with weakly coordinating counter ions PF_6_^-^ and F_3_CSO_3_^-^.

#### 2.1.1. 1D-{[Ag(L_1_)PF_6_]}_n_ coordination polymer **6**

**Figure 5:**
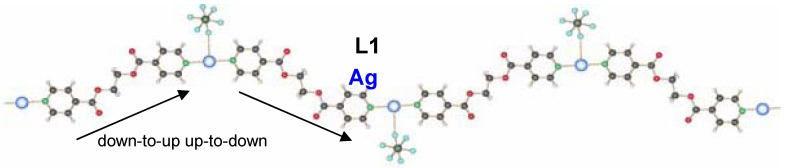
Excerpt of the 1D-chain structure of {[Ag(L_1_)PF_6_]}_n_ **6**.

The compound **6** crystallizes in the monoclinic space group C2/c (No. 15) [17]. The asymmetric unit contains two independent ligand molecules with a C_2_-axis located in the middle of the C–C bond of the ethylene spacer, a silver cation and a hexafluorophosphate counter ion. The two ligands coordinate the silver cation almost symmetrically (Ag(1)–N(1), 2.163(6) Å, Ag(1)–N(2), 2.132(6) Å) with an N(1)–Ag(1)–N(2) angle of 178.9(1)°. The counter ion is a weak coordinating agent; distances F–Ag(1) are for one counter ion 2.903(5) Å (F(1)–Ag(1)) and 3.091(5) Å (F(2)–Ag(1)) for the next closest PF_6_^-^. This linear coordination of the ligands is expected for a poor coordinating ability of the counter ion. The ligand adopts the anti conformation, running alternating up and down as indicated on Figure 5.

#### 2.1.2. 1D-{[Ag(L1)SO_3_CF_3_]}_n_ coordination polymer **7**

**Figure 6:**
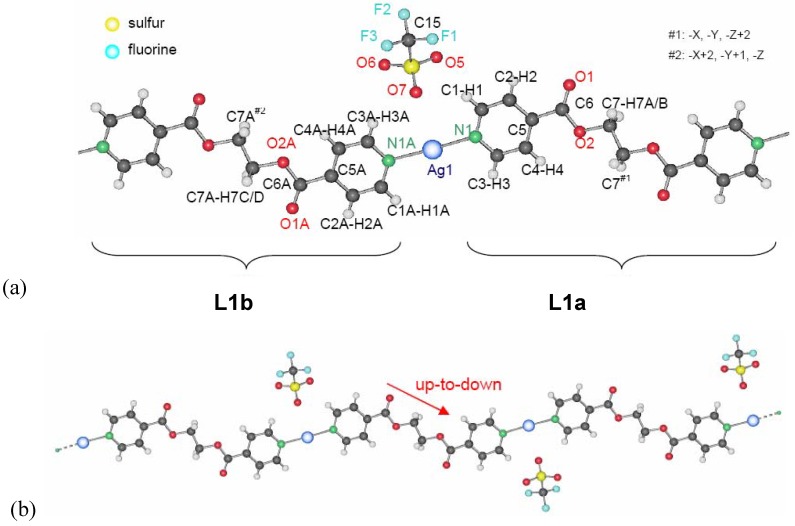
(a) Excerpt of the one-dimensional chain structure of **7** and numbering; (b) ligand orientation in **7**.

The coordination polymer based on silver triflate and L1 crystallizes in a “H-shaped” tube in the darkness at room temperature: a solution of L1 in THF diffuses into a solution of AgCF_3_SO_3_ in water [17]. After one day, crystals of {[Ag(L)]CF_3_SO_3_}_n_ **7** appear at the interface THF/water. **7** is also obtained as a white, polycrystalline powder via the direct reaction of L1 and AgCF_3_SO_3_ in dichloromethane. Its powder X-ray spectrum and the theoretical spectrum for **7** (calculated from the single crystals X-ray data) show that the two compounds are identical, crystallizing in the monoclinic space group P2_1_/c (No. 14). Two half ligand molecules, a counter ion and a metal atom, are contained in the asymmetric unit, the unit cell containing four asymmetric units. As shown in Figure 6, there are two types of ligand molecules in the structure: *i)* the one numbered from N1 to N1#1 (symmetrical equivalent) called L1a; *ii)* the second numbered from N1A to N1A#2 called L1b.

Ag(I) is coordinated linearly by two non-equivalent ligands with very similar distances, **L1** (Ag(1)–N(1) 2.152(5)Å) and **L1A** (Ag(1)–N(1A) 2.154(5)Å), forming an angle N(1)–Ag(1)–N(1A) of 177.4(1)°. In contrast to **6**, the orientation of the ligand molecules within one chain of **7** is always the same, represented as “up-to-down” in Figure 6b.

**Figure 7:**
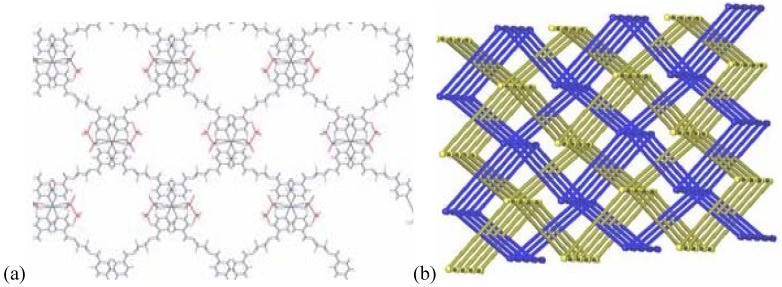
(a) 3D-net of **7** showing large cavities; (b) interpenetrating nets in compound **7**.

The counter ion coordinates in a mono-dentate way to the metal atom Ag(1)–O(5)#1 (2.801(5) Å). At the same time, this counter ion coordinates weakly to a second metal atom of chains running perpendicular above or below the first chain (distance Ag(1)–O(7) of 2.822(5) Å). This way, a three-dimensional network of criss-crossing chains is formed with cavities of 13.183(1) × 14.262(2) Å^2^ (Figure 7a). A second, identical network interpenetrates this first one to fill these channels, running parallel to the first, but displaced by 0.43(2) Å in the *c-*direction. The final structure consists of a 3D framework (classified as Ia, Z = 2 using TOPOS) with Ag(I) cations acting as nodes. These two interpenetrated nets possess a final network topology which is similar to the one present in the CdSO4 salt (Figure 7b).

Among all isolated Ag-coordination polymers so far, with more coordinating anions like NO_3_^-^ and less coordinating anions, only one structure was isolated presenting the ligand in the gauche conformation. Independently of the anions, the 1D coordination polymer motif found that two ligands connect two silver ions forming N–Ag–N-angles of 165–180°, for all compounds **1**–**7**. The arrangement of the ligands with respect to each other within the chain, as well as the orientation of the chains in the crystal, are however very different, reaching from simply parallel packed chains to double chains with metal-metal contacts or interpenetrated arrangements. Among structures with the same counter ion, e.g., nitrate, the Ag–O(NO_3_^-^)-distances were shown to increase in the solid state, the better the solubility of AgNO_3_ in the used solvent. At the same time, the coordination behavior of nitrate is difficult to predict in advance, and renders structure design challenging for such cationic coordination polymers. Such drawbacks can be overcome using e.g., anionic ligands.

In order to render the ligand longer, we added a C_2_H_4_O-group in the middle part of the ligand to obtain L2. The coordination compounds obtained so far with this ligand and silver salts are highlighted in the next chapter and completed with new compounds to gain more insight into the structural variations under the influence of solvent and counter ion.

### 2.2. Compounds with L2

**Figure 8:**
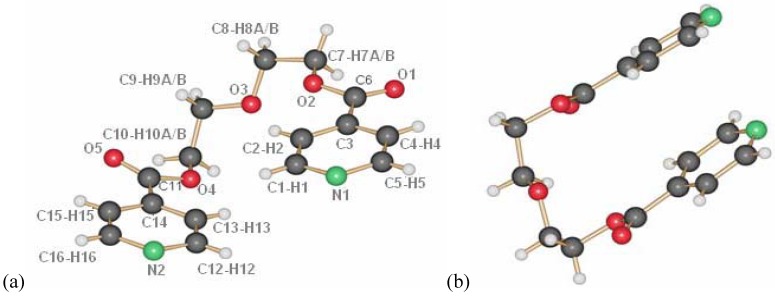
Ligand L2, numbering (a) and U-shape (b).

The ligand L2 is longer than L1 by one ethylene oxide unit. In contrast to ligand L1, it adopts by itself an all gauche conformation with an overall “U”-shape in the solid state (Figure 8a and b). The molecules of L2 are arranged such as they possess π-π-interactions between the pyridine moieties of different molecules. Further details on the ligand have been described previously [18,19].

#### 2.2.1. {[Ag(L2)](ClO_4_)}_2_ (**8**) and its polymorph (**9**)

**Figure 9:**
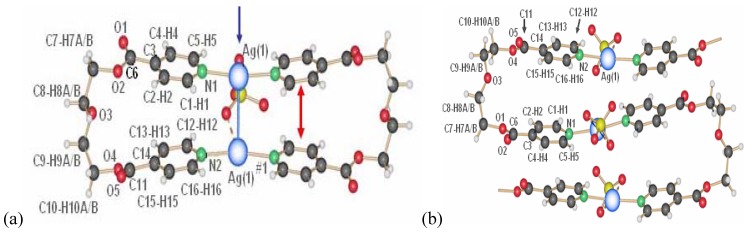
(a) metalla-cyclic compound **8** with short metal-metal-contacts and π-π-interactions; (b) helical chain structure of **9**.

The compound {[Ag(L2)](ClO_4_)}_2_ **8** forms dimeric metalla-cycles, the monomers of which contain one ligand, one silver cation and one perchlorate counter ion (Figure 9a) [18,19]. From the same batch, a one-dimensional helical chain polymorph **9** (Figure 9b) is isolated, exemplifying the possible structural variability of the silver coordination compounds obtained with the same ligand and counter ion [18]. In both compounds **8** and **9**, the diethylene glycol used as spacer presents, similar as the ligand L2 alone, all its ethyl groups in a “gauche” (staggered) conformation. As defined by the term polymorph, the two structures share the same coordination motif around the metal atom: two nitrogen atoms belonging to the pyridine rings coordinate the cation almost in a linear fashion.

The ring-compound **8** presents short Ag–Ag#1 contacts of 3.146(8) Å between silver atoms in the same metalla-cycle, while compound **9** does not have such short metal-metal contacts.

In order to obtain one or the other of the two polymorphs, slight excess of either silver salt (leads to rings) or ligand L2 (leads to the helix) is required and can be realized in a crystallization process in H-tubes.

We then studied the influence of other counter ions on the system. As for the results obtained with ligand L1, coordination compounds of L2 with silver nitrate yielded several different structures depending on the used solvents.

#### 2.2.2. {[Ag(L2)](NO_3_)•2H_2_O}_2_ (**10**), {[Ag(L2)](NO_3_)}_2_ (**11**) and {[Ag(L2)](NO_3_)}_2_ (**12**)

**Figure 10:**
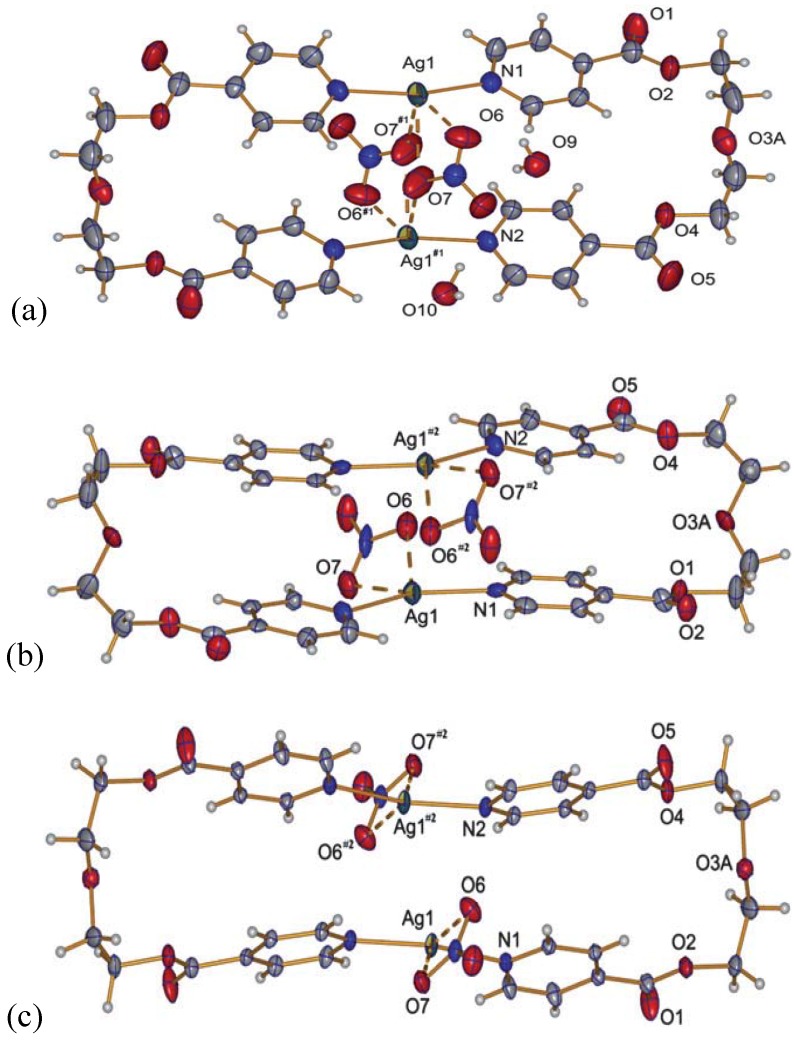
Metalla-cycles formed in compounds **10** (a), **11** (b) and **12** (c).

With nitrate as a counter ion, three different ring structures were obtained and reported previously [20]. In order to show the complete panoply of compounds obtained with L2, the structures of these metalla-cyclic compounds will briefly be reviewed. All three compounds consist of metalla-cycles similar to the one found in compound **8**, but with different influences of the nitrate anions, depending on the solvent from which compounds were isolated. The nitrate anion and its multiple coordinating fashions lead to very different arrangements of the metalla-cycles in the solid state (Figure 10).

Four water molecules co-crystallize per metalla-cycle in {[Ag(L_2_)](NO_3_)·2H_2_O}_2_ **10** when the product of the reaction of L2 with AgNO_3_ is crystallized from a mixture of water and THF [20]. The ring presents with 3.3206(9) Å the shortest metal-metal contact of the three nitrate compounds, and indeed, the two nitrate anions bridge the silver ions of the same ring, forming H-bonds with the water molecules. Despite the short Ag–Ag distance, no aromatic stacking of the pyridine rings is observed because of the tilt angle which they present with respect to each other. Weak inter-ring aromatic stacking interactions are however present at a distance of 3.763(2) Å. The metalla-cycles are stacked in a fishbone motif along the *a*-axis, and the water molecules may be directly responsible for this conformation.

Single crystals of the water-free compound {[Ag(L2)](NO_3_)}_2_ **11** are obtained from THF as only solvent [20]. Like compound **10**, it forms a metalla-cyclic compound, the nitrate anions now coordinating each to only one silver cation in an asymmetric fashion (Figure 10b). Short intra- and inter-metalla-cycle Ag…Ag distances are present, with 3.332(3) and 3.402(3) Å, respectively. The presence of π-π aromatic stacking interactions is excluded due to the distance of 4.007(7)Å between ring centroids.

**Figure 11:**
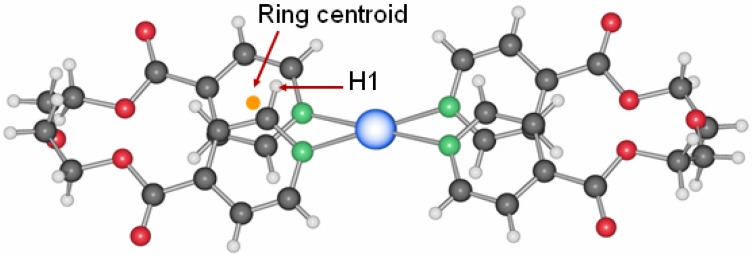
Aromatic interactions in the metalla-cycle system of compound **12**.

An isomer to **11**, {[Ag(L2)](NO_3_)}_2_ **12**, was obtained from DMSO, resembling compound **11**, but with each nitrate anion now coordinating to one silver ion in a symmetrical fashion [20]. Intra- and inter-ring metal-metal contacts are longer than in the two previous compounds with 3.4339(9) Å and 3.648(3) Å, respectively. A short contact of ca. 3.1 Å between a C-H group of one pyridine ring to the mean plane of the other pyridine moiety of the same ligand (Figure 11) indicates aromatic interactions similar to the benzene dimer in the gas phase [21].

#### 2.2.3. {[Ag_2_(L2)_2_](NO_3_)(ClO_4_)}(H_2_O) (**13**)

A curious compound is obtained in the case where, initially by accident, AgNO_3_ and AgClO_4_ were used as starting materials together with L2. A ring compound similar to a fusion of compounds **8** and **10** is obtained, which contains both anions per ring to give {[Ag_2_(L2)_2_](NO_3_)(ClO_4_)}(H_2_O), **13**.

**Figure 12:**
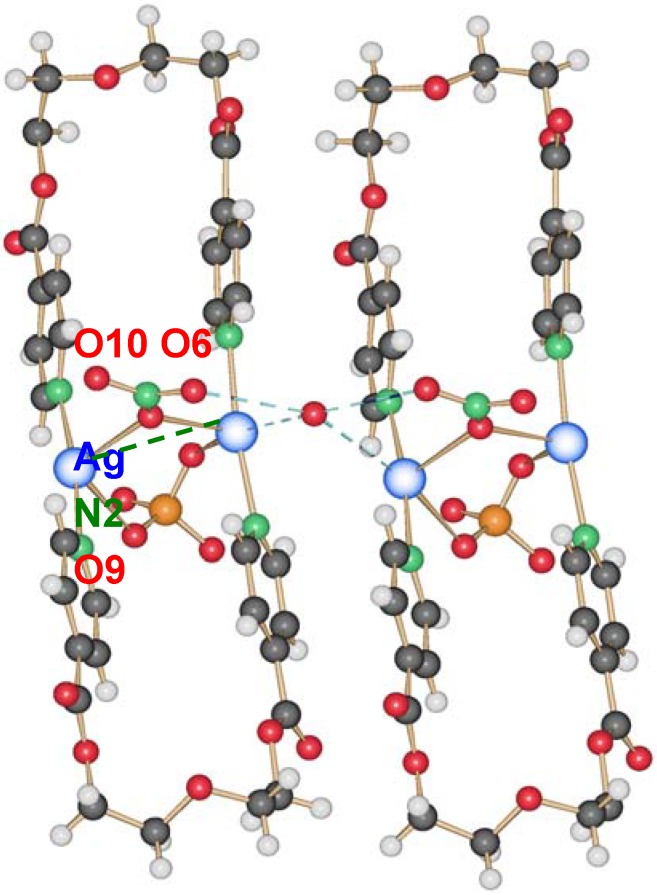
Interaction between two rings of compound **13**; light blue dotted lines indicate H-bonding and weak coordination to silver ions of O10; the green dotted line indicates Ag–π interactions.

This compound crystallizes in the orthorhombic space group P2_1_2_1_2 with one water molecule, one silver ion, one ligand molecule and half of each, a nitrate and a perchlorate anion per asymmetric unit. The two silver ions are coordinated by the two ligands with Ag–N distances of ca. 2.17 Å, a normal range for this kind of interaction, forming a nearly linear N–Ag–N angle of ca. 173.5°. The intraring Ag–Ag contact is with more than 3.5 Å out of range of a metal-metal bond. The nitrate, as well as the perchlorate anion, act as bridging ligands between the silver cations with Ag–O6(nitrate) of 2.624(4) Å and Ag–O9(perchlorate) of 2.861(4) Å, one above and the other below the mean ring plane, indicating the different coordination strength of both anions. One water molecule O10 connects between two rings by forming hydrogen bonds as H-donor with O7 from the nitrate anions, and acting as weak donor ligand between two silver ions (Ag–O10 3.038(3) Å). The packing of the rings is such that the pyridyl moieties of one ligand of a first ring are placed near a silver ion of the next ring, allowing for Ag–N2(π) distances of *circa* 3.4 Å. The general interaction between two neighbor rings is illustrated in Figure 12.

In order to complete the series of compounds obtained with L2, we studied the coordination compounds of this ligand with a rather non-coordinating anion, F_3_CSO_3_^-^ and PF_6_^-^, for which we obtained in total three compounds, two of which are again metalla-cyclic species similarly to **10**–**12**.

#### 2.2.4. {[Ag(L2)](SO_3_CF_3_)}_2_ (**14**)

**Figure 13:**
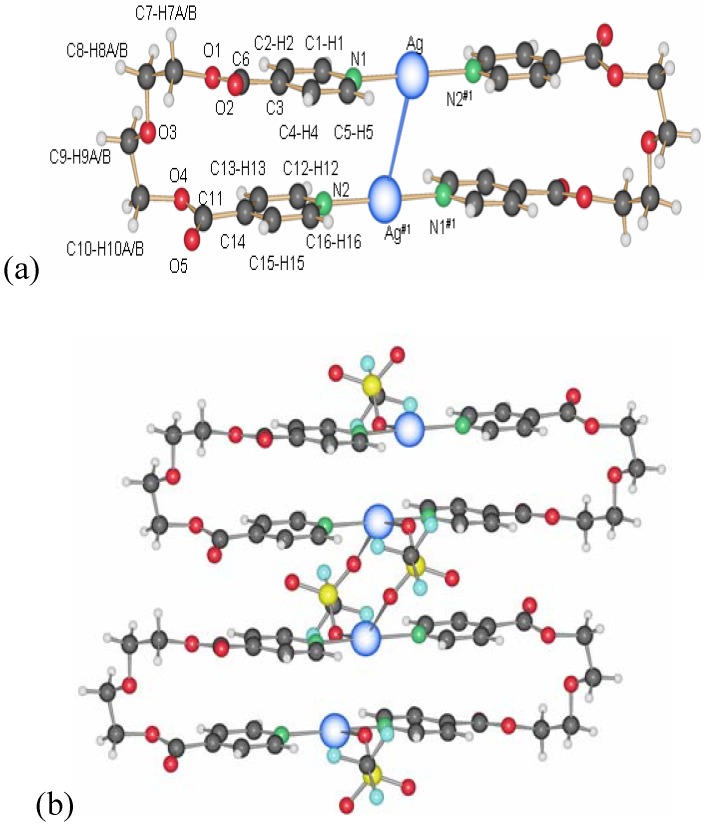
(a) Ring structure of compound **14**; (b) inter-ring bridging of the anions in **14**.

Complex **14** crystallizes in the monoclinic space group P2_1_/c (No. 14), with one ligand and one counter ion coordinating a silver cation in the asymmetrical unit cell [17]. The motif remains the same as before, the metalla-cycle is formed after two ligand molecules adopt a U-shape to coordinate two Ag-ions (Figure 13). Both pyridine rings around a metal ion are arranged almost at the same distances to the cation (Ag(1)–N(1), 2.161(4) Å and Ag(1)–N(2)#1, 2.167(4) Å).

The metal-metal distances are the longest in this series of rings so far with 3.576(8) Å within the ring and 3.832(9) Å between two neighbor rings. The counter ions are accommodated in the spaces formed by four closest ring in a plane, and coordinate two silver atoms from the two neighbor metalla-cycles.

#### 2.2.5. {[Ag(L2)_2_](PF_6_)} and {[Ag(L2)](PF_6_)·THF}_2_ (**15**)

**Figure 14:**
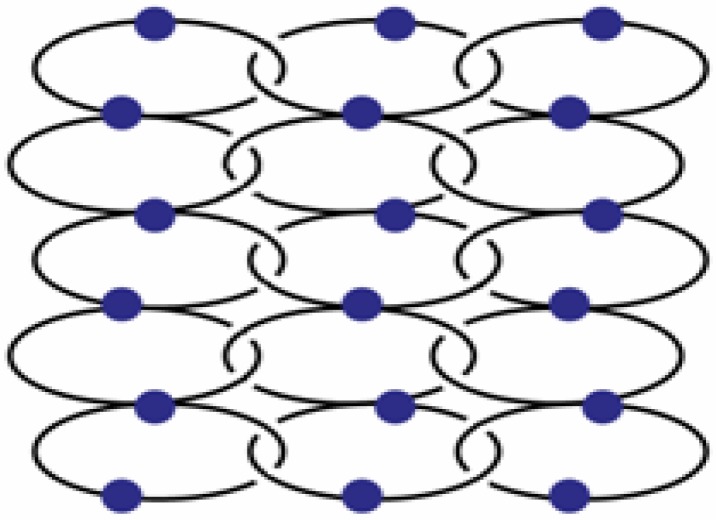
Schematic representation of {[Ag(L2)_2_](PF_6_)}.

**Figure 15:**
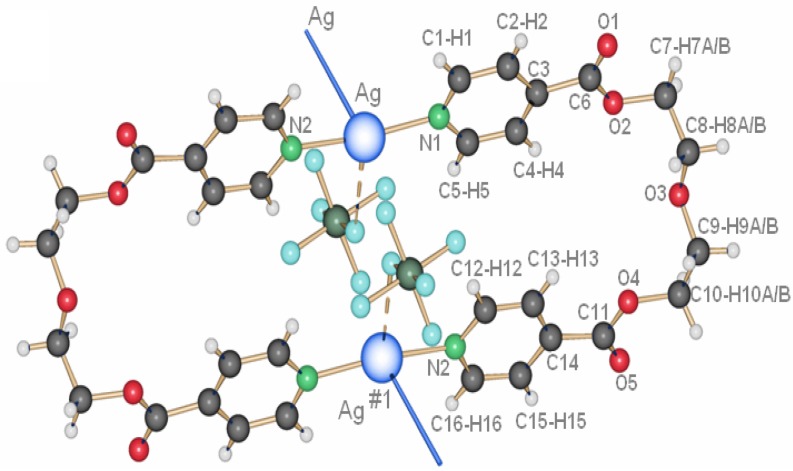
Metalla-cycle obtained for compound **15**.

In the silver salt containing side of the H-shaped tube with water as solvent for AgPF_6_ and THF for L2, two new compounds are obtained [22]. On the ligand-rich side of the H-tube, of {[Ag(L2)_2_](PF_6_)} is obtained, which forms a polycatenated structure (Figure 14) based on interlaced, Ag-fused rings, a structure which has been described previously in detail. This remarkable structure has a ligand to metal ion ratio of 2:1. On the side of the silver salt of the H-tube, the compound {[Ag(L2)](PF_6_)·THF}_2_, **15** is obtained [22]. As for the previous ring compounds, two ligand molecules adopt a “U”-shape and bind to two silver ions to form a metalla-cycle with the anions bridging the two metal-ions (Figure 15). The nitrogen atom of the pyridine ring strongly coordinates the silver cation with Ag–N distances of 2.136(4) Å (Ag(1)–N(2)#1) and 2.143(4) Å (Ag(1)–N(1)). The hexafluorophosphate counter ions are less coordinating towards the metal atom than nitrate ions, which is reflected in the N(1)–Ag(1)–N(2)#1 angle of 169.3(2)°. The Ag–Ag distance within the ring is rather long with 5.213(1) Å, and π-stacking within the ring is thus excluded with pyridine rings offset by *circa* 5.1 Å to each other. All diethylene glycol moieties posses a “gauche” (staggered) conformation with torsion angles of about 67.2(4)° (O_2_–C_7_–C_8_–O_3_) and -70.3(4)° (O_3_–C_9_–C_10_–O_4_).

Among all compounds obtained with ligand L2, the ligand, in contrast to L1, preferred the all gauche conformation to yield metalla-cyclic species, except in one case, where, still in gauche conformation, it yields a 1D-coordination polymer. The metalla-cyclic entities formed of two metal ions and two ligand molecules, are completed by the counter ions. These play the role of intra- or inter-molecular bridging ligands. As for compounds with L1, the nitrate ion showed the most different coordination possibilities, yielding in different assemblies of rings. These arrangements also induce different metal-metal distances, which influence the light stability of these compounds [24,26,27,28]. The closer the metal ion contact, the easier is the compound reduced with light.

### 2.3. Theoretical Calculations

For all of our six homoanionic ring compounds, **8** (ClO_4_), **10**–**12** (NO_3_), **14** (SO_3_CF_3_) and **15** (PF_6_), we carried out electronic structure calculations in order to compare with experimental structures and to understand the individual contributions of selected forces in the ring systems [20]. Thus, DFT calculations at the B3LYP/6–311++G** level [20 and therein] were carried out to estimate the stabilization energy due to ligand L2 in the metalla-cycles. The crystallographic coordinates of the organic ligand were extracted from the crystal structures of L2:Ag 1:1 complexes (Lc) and used in the theoretical model without further optimization. The calculated value from the crystallographic data of the crystal structure of the ligand L2 was used as a reference. All data obtained are expressed as the difference between L2 and Lc. The results obtained show the total energy of the ligand in the respective compound (ΔE_total_), the energy due to the π-π-interactions (ΔE_ring_) and stabilization energy due to the diethylene glycol conformation (ΔE_torsion_) (Table 1).

**Table 1:** DFT calculation results for the ligand in the metalla-cycles, all calculated energies are given in kcal/mol.

Compound	ΔE_total_	ΔE_ring_	ΔE_torsion_	π-π (Å)	torsion ang.(°)
**L2**	---	---	---	5.801(6)	69.9(2), 62.6(6)
**8**, ClO_4_	-28.6	-20	-8.2	3.704(5)	52.7(1), 67.7(4)
**10**, NO_3_	+10	-7.2	+16.5	5.142(6)	46.1(4), 79.3(6)
**11**, NO_3_	+20.1	-5.1	+25.4	4.082(9)	73.2(9), 15.4(2)
**12**, NO_3_	+74.1	+3	+68.7	4.149(8)	62.3(9), 50.8(2)
**14**, SO_3_CF_3_	-3.9	-5.8	+1.4	3.965(4)	62.7(1), 68.1(1)
**15**, PF_6_	-6	-7.6	+0.8	5.775(8)	67.2(5), 70.2(7)

**Figure 16:**
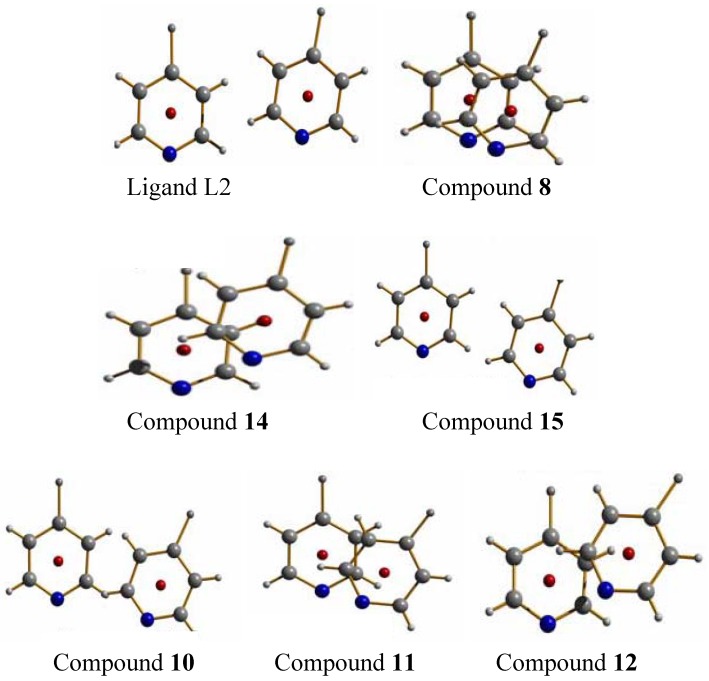
Relative positions of the pyridyl units of the ligand L2 of the different compounds (centroids in red).

As expected, the ΔE_ring_ depends on the distance between the aromatic ring centroids and the position at which the pyridine rings are stacked (Figure 16). Approaching the centroids of the two aromatic rings may increase the repulsion between the two moieties due to electron repulsion. This is proportional to the values found for ΔE_ring_. Clearly, along with the short Ag–Ag distance, compound **8** presents the shortest distance between the pyridine moieties and is consequently the most destabilized compared to the free ligand L2. In compound **12**, a stabilizing effect compared to the free ligand is probably due to the favorable C-H/π type of interaction discussed above.

Concerning the diethylene glycol chain, the torsion angles of compounds **10** and **11** are relatively constrained compared to the values found in the organic ligand of other metalla-cycles. This fact could explain in part the energy difference, which may significantly destabilize the rings in both these compounds compared to the other metalla-cycles in this series. The **ΔE total** is calculated without taking into account the ligand-silver(I) and silver(I)-counter ion interactions in the metallacycle, thus, this result concerns only the ligand part of the ring. Also, it does not take into account inter-ring interactions. Yet, it is noteworthy that the value of **ΔE total** is approximately equal to (**ΔE ring + ΔE torsion**).

### 2.4. Applications of Silver Coordination Compounds

#### 2.4.1. Antimicrobial Properties

Silver compounds have been known for a long time to possess antimicrobial properties. Thus, our compounds were tested in this context for their bactericidal properties on one hand, and for their biocompatibility on the other. Being a review, only the general qualitative trends of these studies will be described here, while details have been published elsewhere [23,24,25].

**Figure 17:**
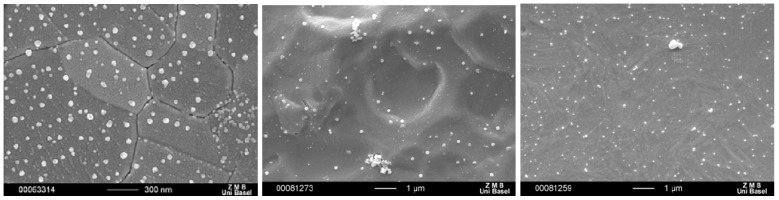
SEM image of nanostructures of compound **1** on Au(111) (left), on rough Ti (middle) and on rough steel (right).

In a first step, we have deposited coatings of the above silver compounds, namely **1**, **2**, **11** and **12**, on metallic substrates such as gold alloy as used in dental applications, titanium and steel as used for implants or fracture fixation devices, and a monolayer of gold, Au(111). This latter substrate allowed us to carry out model studies, learning about the type of nano-structured depot that is formed on an implant material by the different compounds. All four studied compounds were able to form nano-structured depots on gold and titanium by inserting the substrates into a mother liquor of the freshly prepared compounds (e.g., Figure 17), followed by powder x-ray diffraction for identification [26,27,28].

**Figure 18:**
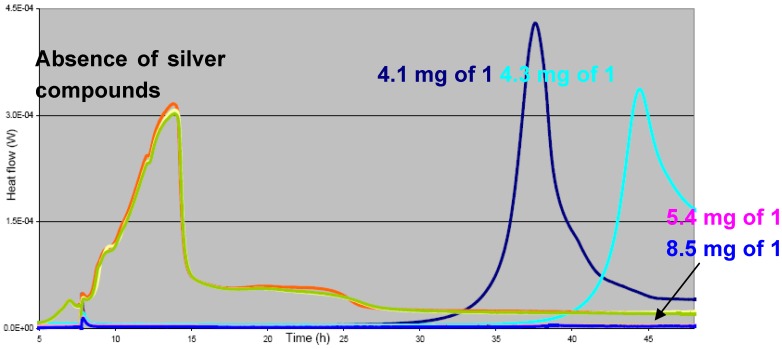
Heat flow curves for *S. epidermidis* in the absence (green, yellow, orange) and presence (dark blue, light blue, medium blue, pink) of different quantities of compound **1**.

As a general trend, it was found that–from similar substrate loadings–the solubility of the ring structures **11** and **12** was generally higher than the ones of the coordination polymers **1** and **2**, releasing therefore more silver ions into the surrounding medium [26,27]. For compounds **1** and **2**, the released silver concentration is in the order of 1–10 ppb, while for the rings **11** and **12**, it is 4.2 ppm for a one-day coating duration. The bacterial inhibition potential of our coated samples was assessed first by micro- and nano-calorimetry and showed that in presence of our silver compounds, the onset of bacterial growth is strongly delayed if not abolished compared with the multiplication of bacteria in absence of antimicrobial agents (Figure 18). This effect is dependent on the type and quantity of silver compounds present [26,27].

**Figure 19:**
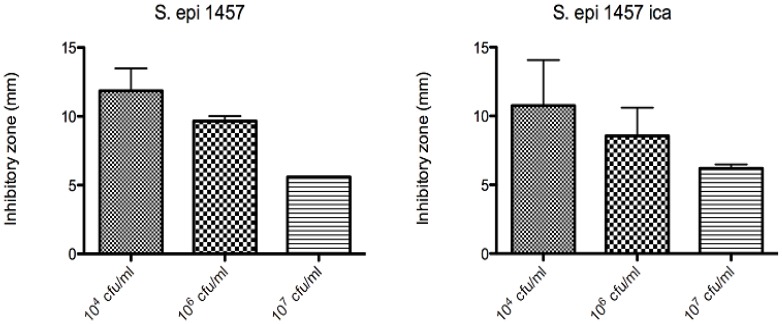
Inhibition zones for Au(111)-samples coated with compound **11** (10^-3^ mol/L of **11**, coating duration of one day) at different starting concentrations of *S. epidermidis 1457* (left) and *S. epidermidis 1457* depleted of *ica* (biofilm forming gene) [26,27].

The antimicrobial activity was also investigated by Kirby-Bauer-tests for *S. epidermidis*, *S. aureus*, *E. Coli* and *P. aeruginosa*. In our samples, the inhibition zones reached up to *circa* 1.5 cm in diameter for the polymer **1** [27] and up to 1.2 cm for the ring compound **11**, both of which is a signature of a very efficient antimicrobial agent (Figure 19).

**Figure 20:**
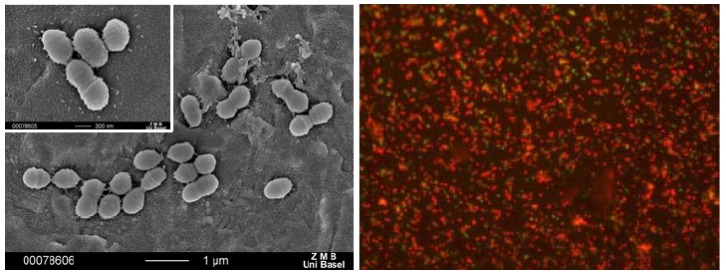
Bacterial adhesion to gold alloy (left) and optical micrograph of dead bacteria (with fluorescent marker) on a gold alloy coated with compound **1** (right).

We have also coated dental restorative materials such as a gold alloy with compound **1**. In a flow chamber imitating the oral cavity [24], it was shown that bacteria might still adhere to such a coated surface, but that these bacteria are killed upon contact with the substrate (Figure 20).

**Figure 21:**
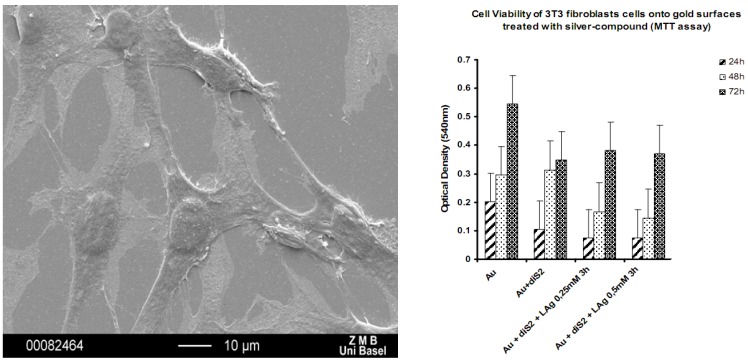
SEM of mouse fibroblasts adhering to a with **1** coated Au(111) substrate (left); cell viability of mouse fibroblast cells as a function of substrate coating [29].

The silver concentration due to silver ion release from the coating of **11** and **12** was so high that also eukaryotic cells such as fibroblasts were damaged (results not shown) [26,27]. Thus, the ring compounds are not suitable for being used as implant coatings because of lacking biocompatibility. The coordination polymer compounds however were tested for their compatibility with fibroblasts and osteoblasts and turned out to be the ideal candidates for such antimicrobial coatings. Indeed, we could show via SEM, MTT and DAPI staining tests that both, fibroblasts and osteoblasts are i) alive on our substrates and ii) proliferate as well (Figure 21) [29].

#### 2.4.2. Formation of Nanoparticles

**Figure 22:**
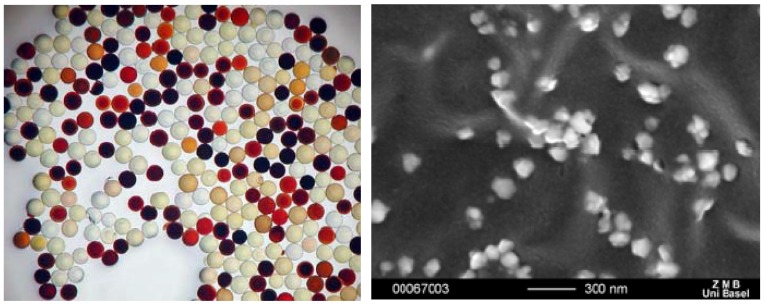
Styrene beads loaded with silver nanoparticles as a function of the amino acid sequence (left) and SEM of nanoparticles on red beads (right).

While the formation of gold nanoparticles is now well established, it is commonly more difficult to stabilize silver nanoparticles at a given size. Thus, in parallel to the antimicrobial properties of the silver coordination compounds, we studied the interaction of silver ions with short peptide sequences using a split and mix library as described previously in order to learn about the interaction of the metal ions with larger biomolecules as well as the potential ability of these amino acids to reduce silver. [11,12] Indeed, depending on the amino acid sequence, it was found that certain sequences are able to bind more or less silver ions and possess different abilities to reduce silver ions to silver nanoparticles [11,12,30]. Thus, the size of the formed nanoparticles varies with the amino acid sequence (Figure 22).

## 3. Experimental Section

Synthesis of Compounds: All chemicals and solvents were used as received from Fluka or Aldrich. Only the new compounds are presented here, the reader may refer to the original papers for those previously published.

Compound **6**: In an H-shaped tube, 30 mg L1 (0.11 mmol) was dissolved in 10 mL THF in one arm of the tube while 27.8 mg AgPF_6_ (0.12 mmol) was dissolved in 10 mL distilled water in the other side. Both compartments were bridged by a mixture of both solvents. Pale green crystals were picked from the wall of the H-shaped tube after *circa* 2 weeks. Crystals formed in both sides of the H-shaped tube afford the same complex. Other changes in the ratio ligand/silver salt (1:2 or 2:1) afford the same complex. Yields (**6**): 40.2 mg of **6** (0.076 mmol, 64% calculated with respect to AgPF_6_). Elemental analysis: calculated: C 32.0, H 2.30, N 5.34%; found: C 32.32, H 2.38, N 5.20%. IR (cm-1): ν(Ar-H) 3112.3 s, ν(-HC-H) 3986.6 s, ν(C=O) 1605.4 s, ν (ArC–C, C=N) 1447.9 m, ν(C–O) 1219.2 m, δ(ArC–H) 1072.3 m, ν (PF6) 828.5 s, broad.

Compound **7**: A solution of silver triflate (20 mg, 0.07 mmol) and L1 (21.25, 0.07 mmol) was refluxed in the microwave (580 W) for 4 minutes. The hot solution was filtered and the solution was left at room temperature for three days. Single crystals of complex **7** were obtained in the wall and the bottom of the vessel after slow evaporation. The same compound is obtained by diffusion in an H-shaped tube, but requires more than 20 days to crystallize. Yields (**7**): 29.7 mg of **7** (0.056 mmol, 80% calculated with respect to AgCF_3_SO_3_). Elemental analysis: calculated: C 34.03, H 2.29, N 5.29%; found: C 34.5, H 2.3, N 5.27%. IR (cm^-1^): ν(Ar-H) 3075.5 s, ν(-HC-H) 2957.7 s, ν(C=O) 1721.9 s, ν(C=C) 1604.1 m, ν(ArC–C,C=N) 1423.6 m, ν(SO_3_CF_3_) 1273.7 s, broad, ν(SO_3_CF_3_) 1108.8 s, broad, δ(ArC–H) 1072.3 m.

Compound **13**: Typically, an equimolar mixture of AgNO_3_ and AgClO_4_ was dissolved in water and placed in one arm of an H-tube, while ligand L2 was dissolved in THF to give a ratio 1:1 with silver ions and placed in the other arm. Slow diffusion through the connecting bridge and THF leads to the formation of single crystals of **13**. Yields (**13**): 25% calculated with respect to Ag^+^). Elemental analysis (13): calculated: C 37.39, H 3.39, N 6.81%; found: C 37.22, H 3.25, N 6.77%. IR (cm-1): ν(Ar-H) 3090.2 s, ν(-HC-H) 2953.8 s, ν(C=O) 1722.9 s, ν(C=C) 1609.8 w, ν(ArC–C,C=N) 1428.1 m, ν(NO_3_) 1391.5-1187.2 s, ν(ClO_4_) 1100 s, broad.

Compound **14**: Silver triflate (20 mg, 0.07 mmol) was dissolved in water (5 mL), and the solution was connected with THF to a solution of THF (5 mL) containing L2 (22.12 mg, 0.07 mmol). Single crystals were formed after some weeks on the bottom of both sides in the H-shaped tube. The complex **14** was obtained in the side of the ligand. Yields (**14**): 22.6 mg of **14** (0.042 mmol, 60% calculated with respect to AgSO_3_CF_3_). Elemental analysis (**14**): calculated: C 34.03, H 2.29, N 5.29%; found: C 34.0, H 2.22, N 5.2%. IR (cm^-1^): ν(Ar-H) 3102.2 s, ν(-HC-H) 2933.8 s, ν(C=O) 1723.9 s, ν(C=C) 1619.8 w, ν(ArC–C,C=N) 1431.1 m, ν(SO_3_CF_3_) 1260.0 s, broad, ν(SO_3_CF_3_) 1092–1039 s.

### Single Crystal Structures

Single crystals were mounted on a glass fiber and all geometric and intensity data were taken from this crystal. Data collection using Mo-Kα radiation (*k* = 0.71073 Å) was performed on a STOE IPDS-II diffractometer equipped with an Oxford Cryosystem open flow cryostat [31]. Absorption corrections were partially integrated in the data reduction procedure [32]. The structures were solved by direct methods (SHELXS) [33,34] and refined using full matrix least-squares on *F*^2^ (SHELXL 97) [33,34]. All heavy atoms could be refined anisotropically. Hydrogen atoms were introduced as fixed contributors when a residual electronic density was observed near their expected positions.

*Compound* **6**: AgC_14_H_12_N_2_O_4_PF_6_, M = 525.09 gMol^-1^, monoclinic, *C*2/c (No. 15); *a* = 35.290(8); *b* = 10.453(2); *c* = 9.382(2)Å; *β* = 101.43(3)°; *V* = 3392.2(12) Å^3^; *Z* = 4; *T* = 203(2)K; 2214 reflections; 2.92° < θ < 24.96°; *F*(000) = 2064; *ρ* = 2.056 gcm^-1^, *μ* = 1.372, 1214 reflections > 2σ; parameters 253; R(all) = 0.0879; R( > 2σI) = 0.0447; wR(all) = 0.0834; wR = 0.0720; GooF = 1.034.

*Compound* **7**: C_15_H_12_AgF_3_N_2_O_7_S, M = 529.20 gMol^-1^, monoclinic, *P*2_1_/c (No. 14), *a* = 8.995(2), *b* = 21.389(4); *c* = 9.490(2)Å; *β* = 95.47(3)°; *V* = 1817.4(6) Å^3^; *Z* = 4; T = 293(2)K, 14456 reflections of which 4008 independent and 2409 > 2σI; R(int) = 0.1077, 1.90° < θ < 27.16°; F(000) = 1048, ρ = 1.934 gcm^-1^, μ = 1.297 cm^-1^, 262 parameters; R(all) = 0.1132, R = 0.0562; wR(all) = 0.1125; wR = 0.0959; GooF = 1.038.

*Compound* **13**: C_32_H_34_Ag_2_ClN_5_O_18_; M = 1027.80 gMol^-1^; orthorhombic, *P*2_1_2_1_2, *a* = 22.684(5); *b* = 7.1516(1); *c* = 11.732(2) Å; *V* = 1903.2(6) Å^3^; *Z* = 4; *T* = 293(2)K; 13623 reflections of which 4041 independent and 3229 observed, R(int) = 0.0589, 3.34 < θ < 26.84°, F(000) = 1028, 265 parameters, R(all) = 0.0636, R = 0.0448; wR(all) = 0.1445; wR = 0.1307, GooF = 0.987.

*Compound* **14**: C_17_H_16_AgF_3_N_2_O_8_S; *M* = 573.25; monoclinic, *P*2_1_/c (No. 14), *a* = 7.0987(14), *b* = 26.081(5), *c* = 11.121(2)Å, *β* = 91.10(3)°, *V* = 2058.6(7) Å^3^, *Z* = 4, *T* = 293(2)K, F(000) = 1144, *μ* = 1.156cm^-1^, 16323 reflections, 4528 independent and 3581 observed, R(int) = 0.0985, 1.56 < θ < 27.13°, 290 parameters, R(all) = 0.0631; R = 0.0480, wR(all) = 0.1201, wR = 0.1307, GooF = 1.069.

CCDC-765996(**6**), 765995(**7**), 765994 (**13**), 765997 (**14**) contain the supplementary crystallographic data for this paper. These data can be obtained free of charge from The Cambridge Crystallographic Data Centre via www.ccdc.cam.ac.uk/data_request/cif. For the other compounds, data have been submitted previously.

Kirby-Bauer-tests: Typically, a pure culture of microorganisms was grown overnight, and then diluted to a concentration of about 1 × 10^6^ microorganisms per milliliter. The diluted microbial suspension was spread evenly over the face of a sterile agar plate by a sterile swab, into which the gold plate (uncoated as reference, coated as our samples) was placed in the center of the agar plate and incubated. If substantial antimicrobial activity is present, then a zone of inhibition appears around the test product. The zone of inhibition is simply the area on the agar plate that remains free from microbial growth. The size of the zone of inhibition is usually related to the level of antimicrobial activity present in the sample or product-a larger zone of inhibition usually means that the antimicrobial is more potent.

## 4. Conclusions

In conclusion, we have highlighted here the different coordination polymer networks and ring-compounds obtained with two ligands, L1 and L2, differing only in the spacer length used to separate the coordinating pyridyl units. Whereas L1 adopts the stretched out conformation (anti) when crystallizing alone as well as when coordinated to metal ions, the ligand L2 seems to preferentially adopt the gauche conformation resembling a “U”-shape. While coordination of the ligands L1 and L2 to silver ions give certain assemblies in a first step (1D-coordination polymer or ring), the arrangement of these entities in the crystal may be very different, and is dependent of anions as well as solvents. Strongly coordinating may give many different compounds. We believe this to be an effect of solubility of the initial metal salt in the chosen solvent. Thus, the better the solvent, the longer is the final metal-anion contacts. For the ring compounds, the role of the anions on the inter- and intra-ring contacts has been studied by a theoretical approach. All in all, the ligand allows tuning of the structure and inherently also of the solubility, which plays an important role in the potential use of silver compounds as antimicrobial agent for the prevention of e.g.m implant infection.
